# Metabolic Syndrome and Framingham Risk Score: Observation from Screening of Low-Income Semi-Urban African Women

**DOI:** 10.3390/medicines3020015

**Published:** 2016-06-09

**Authors:** Ayokunle S. Dada, Daisi D. Ajayi, Peter O. Areo, Taiwo H. Raimi, Eyitayo E. Emmanuel, Olusola O. Odu, Olusegun A. Aremu

**Affiliations:** 1 Department of Medicine, Ekiti State University Teaching Hospital, Ado Ekiti 36001, Ekiti State, Nigeria; adechristie2004@yahoo.com (T.H.R.); aremuolusegun101@hotmail.co.uk (O.A.A.); 2 Department of Medical Laboratory Science, Ekiti State University Teaching Hospital, Ado Ekiti 36001, Ekiti State, Nigeria; ajayiekitilab@yahoo.com; 3 Department of Surgery, Ekiti State University Teaching Hospital, Ado Ekiti 36001, Ekiti State, Nigeria; areolafemoris@yahoo.co.uk; 4 Department of Community Medicine, Ekiti State University Teaching Hospital, Ado Ekiti 36001, Ekiti State, Nigeria; eyitayoe456@gmail.com (E.E.E.); borbordoc4@gmail.com (O.O.O.)

**Keywords:** metabolic syndrome, Framingham risk score, cardiovascular disease

## Abstract

**Background:** The heightened cardiovascular risk associated with metabolic syndrome (MetS) has been documented by several researchers. The Framingham risk score (FRS) provides a simple and efficient method for identifying individuals at cardiovascular risk. The objective was to describe the prevalence of MetS and its association with FRS in predicting cardiovascular disease among a cohort of semi-urban women; **Method:** Clinical and laboratory parameters were evaluated among 189 healthy women. The International Diabetes Federation definition was used to diagnose metabolic syndrome. FRS was calculated for each participant; **Result:** About two thirds of the participant make less than $US 90 per month. The mean systolic blood pressure was 131.80 ± 30. Eighty (42.3%) participants were overweight with a mean waist circumference of 91.64 ± 11.19 cm. MetS was present in 46 (24.3%). Individuals with MetS were more likely to have increased FRS, *p* = 0.012. One hundred and eighty seven (98.9%) were in the low risk category according to FRS. There was a significant difference in the mean FRS between participants with and without MetS (13.52 *versus* 10.29 *p* = 0.025); **Conclusion:** Prevalence of MetS in this study was comparable to the global rate, despite a low economic status. Individuals with MetS were more likely to have cardiovascular disease than persons without MetS, thus emphasizing the need for risk stratification and prompt management.

## 1. Introduction

There is increased reported prevalence of obesity and metabolic syndrome in Sub-Saharan Africa. This may be attributed to the adoption of Western lifestyles, including diet and reduced physical activity. Several clinical conditions are specific to women. Stress associated with pregnancy, use of oral contraceptive medication, polycystic ovary syndrome, and the effects of menopause [1,2]. The aforementioned conditions, in association with traditional cardiovascular risk factors, put women at a greater disadvantage and, hence, are candidates for special considerations regarding risk factor identification, modification, and clinical management [2].

In the developed countries, cardiovascular mortality rates in women have declined due to modifications in risk behavior, such as increased physical activity, better management of hypertension and dyslipidaemia, and improved treatment of existing cardiovascular conditions [3].

These benefits are not apparent in developing countries, where only a fraction of women with cardiovascular disease (CVD) has access to optimal care [4,5].

Metabolic syndrome has been linked with cardiovascular disease. The heightened cardiovascular risk associated with metabolic syndrome has been documented by several researchers. Reports from developing countries showed that cardiovascular disease represents up to 75% of mortality from non-communicable diseases, and accounts for about 10% of the developing world’s burden of morbidity [5,6]. Cardiovascular disease is projected to be a principal cause of death and disability worldwide by 2030 [7,8,9].

Many reports also point out that the presence of metabolic syndrome (MetS) contributes to an increased risk for CVD and type-2 diabetes. A two- and five-fold increase risk has been associated with MetS in the development of CVD and diabetes, respectively [10,11].

The Framingham risk score (FRS) is a gender-based algorithm designed to estimate an individual’s 10-year chance of developing cardiovascular disease [12].

The scoring system is important as it gives an indication of the likely benefits of prevention and prompt management of cardiovascular risk factors. It also provides an economical and efficient method of identifying individuals at high cardiovascular risk who need preventive treatment, and persons with low risk who need not be unduly worried [13].

Many earlier studies in Nigeria described the prevalence of risk factors for CVD in specific populations, such as those with hypertension, diabetes, and the aged [14,15,16]. The aim of the study is to provide descriptive information on the prevalence of metabolic syndrome and its association with the Framingham risk score in predicting cardiovascular disease among a cohort of semi-urban African women.

## 2. Methods and Settings

A cross-sectional pilot study was conducted among Christian women during their annual congress meeting at Ado-Ekiti, Nigeria. The participants had an initial health-awareness talk a day before the screening exercise. One hundred and eighty-nine women voluntarily participated in the screening exercise. The International Diabetes Federation (IDF) definition was used to diagnose metabolic syndrome.

The anthropometry measurements, including the height, weight, and waist and hip circumferences, were taken. Weight was measured using a standardized bathroom scale, while the height was measured with a stadiometer. The waist circumference was taken at mid-way between the sub-costal margin and the iliac crest with an inelastic measuring tape, while the hip circumference was taken at the widest diameter. The body mass index (BMI) was calculated as weight in kilograms divided by the square of height in meters.

BMI status was categorized as follows: Underweight < 18.5; normal 18.5–24.9; overweight 25–29.9; obese ≥ 30.

Trained nurses and doctors took blood pressure measurements from the left upper arm with the participants sitting using Accusson’s mercury sphygmomanometer with appropriate cuff sizes. Fasting total and high-density lipoprotein cholesterol, triglycerides and blood glucose were estimated for all participants. The low-density cholesterol was calculated using the Friedewald formula [17]. All parameters were expressed as mmol/L.

Participants were diagnosed to have diabetes mellitus (DM) if they had a fasting blood glucose level (FBG) ≥ 7 mmol/L, or reported a history of diabetes, or use of glucose-lowering drugs.

Written inform consents were signed by all participants. Ethical clearance was obtained from the ethical review committee of the Ekiti State University Teaching Hospital, Ado Ekiti, project identification code EKSUTH/A67/2014/10/10, 10 October 2014.

The IDF criteria [18] were used to define the presence metabolic syndrome as follows: Central obesity (defined as waist circumference ≥ 80 cm) plus any two of the following four factors:

1. Raised TG level: ≥ 1.7 mmol/L, or specific treatment for this lipid abnormality
2. Reduced HDLchol cholesterol: <1.29 mmol/L in females, or specific treatment for this lipid abnormality
3. Raised blood pressure: Systolic BP ≥ 130 or diastolic BP ≥ 85 mmHg, or treatment of previously diagnosed hypertension
4. Raised fasting plasma glucose (FPG) ≥ 5.6 mmol/L, or previously diagnosed type 2 diabetes.

We use the Sub-Saharan threshold for waist circumference to define central obesity, *i.e.*, ≥80 cm. Participants found to have diabetes mellitus or raised fasting plasma glucose were regarded to have dysglycaemia.

FRS for cardiovascular disease over 10 years was calculated for each participant based on age, gender, total cholesterol, HDL-cholesterol, and systolic blood pressure or currently on any medication to treat high blood pressure [12]. Individuals with a score of 10% or less had a low coronary disease risk at 10 years, while those with 10%–20% and 20% or more were categorized as having intermediate and high risk, respectively.

## 3. Statistical Analyses

Data for a continuous variable were presented as mean (SD), and as number and percentages for categorical variables. Chi-square and student’s *T* test were used for comparison of individuals with and without metabolic syndrome. A *p* value of <0.05 was taken as significant.

## 4. Results

One hundred and eighty-nine women participated in the screening exercise. Their mean age was 53.50 ± 9.64, with a range of 32–80 years, and about two thirds (58.2%) were between 46 and 60 years. About equal proportions were civil servants and petty trader 41.3% and 39.2%, respectively, while 16 (8.5%) practiced subsistence farming as an occupation (Table 1).

As illustrated in Table 1, almost equal numbers of participants attained tertiary and secondary education; fifty-nine (31.2%) and 61 (32.3%), respectively. About two thirds of participants (60.3%) live on less than $US 90 per month, while only 4 (2.1%) earn more than $US 500 per month (Table 1).

The mean systolic and diastolic blood pressures were 131.80 ± 30.17 and 78.28 ± 13.58 mmHg, respectively. The mean body mass index (BMI) was 27.11 ± 6.84. The waist circumference, hip circumference and the waist-hip ratio were 91.64 ± 11.19 cm, 105.47 ± 13.64 cm and 0.87 ± 0.07, respectively.

The mean fasting blood sugar was 5.40 ± 1.76 mmol, while the values of fasting lipid profiles were as follows: Total cholesterol 5.43 ± 1.06 mmol/L and HDL-cholesterol 1.62 ± 0.47 mmol/L, TG 1.14 ± 0.46 mmol/L, LDL-cholesterol 3.28 ± 0.48 mmol/L (Table 2).

Fifty-three (28.0%) were either on antihypertensive medications or had systolic BP ≥ 130 or diastolic BP ≥ 85 mmHg, while 22 (11.6%) had fasting glucose level diagnostic of diabetes mellitus or on medication for diabetes mellitus. Eighty (42.3%) participants were overweight, while 55 (29.1%) and 6 (3.2%) had normal BMI and were underweight, respectively. The proportions of mild, moderate and severe obesity were 33 (17.5%), 10 (5.3%) and 5 (2.6%), respectively.

Metabolic syndrome was present in 46 (24.3%) of the participants. Individuals with metabolic syndrome were significantly more likely to have increased FRS, *p* = 0.012. Overall, 187 (98.9%) were in the low-risk category and only 2 (1.1%) were in moderate risk category group. Among those with metabolic syndrome, 44 (23.3%) had 10-year cardiovascular risk <10%, only 2 (1.1%) had 10%–20% risk, and none were in the high-risk category according to FRS.

**Figure 1:**
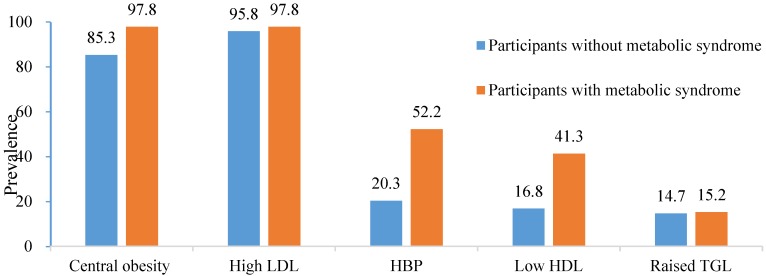
Prevalence of individual components of metabolic syndrome in the entire study population and in the participants with metabolic syndrome.

There was a significant difference in the mean FRS between participant with and without metabolic syndrome (13.52 *versus* 10.29 *p* = 0.025). Among the participants with metabolic syndrome, central obesity and high LDLchol were the prevalent disorders (Figure 1).

Within the 45–60-year age group, 110 (58.8%) were in the low-risk category, while a lower prevalence (19.8% and 21.4%) was found in the 31–45 years and >60 years age groups, respectively.

## 5. Discussion

Women suffer from the pandemic of cardiovascular diseases (CVD). It has been documented that heart disease is the leading cause of death in women in resource-rich countries, with a worse outlook in developing countries [19]. CVD is often thought to be a disease of affluence, but mortality rates in women over the age of 60 are more than double in low- and middle-income countries than in high-income countries [20].

The first women-specific clinical recommendations for the prevention of CVD were published in 1999 [21], with a modification/reviewed in 2007. The guidelines emphasize the importance of pre-clinical detection of disease to identify asymptomatic individuals at high risk, with the sole aim of benefit from early intervention [22].

The prevalence of metabolic syndrome in this study was 24.3%. This is comparable to the 26% reported in a survey conducted among urban Asian Indians [23], and the report of Ogbu *et al.* from Nigeria [24].

A systematic review designed to estimate the overall distribution of Cardiometabolic syndrome in Nigeria by Oguoma *et al.* reported a prevalence of 28.1% using IDF criteria [25]. Similar prevalences were documented in reports from other studies [26,27,28,29].

The present study contrasts with the report of Adediran *et al.*, who reported 7.7% and 14.9% prevalence rates of metabolic syndrome among rural and urban dwellers, respectively, in Abuja, Nigeria [30]. Olajire *et al.* reported the prevalence of metabolic syndrome to be 11.8% among the participants from three rural towns in Southwestern Nigeria [31].

In rural areas, the prevalence of the syndrome remains considerably lower, as reported by many studies. People with a traditional lifestyle in rural communities engage in daily physical activity and consume less energy-dense foods [30,32]. In addition, the observed discrepancy in the various studies might be due to differences in metabolic syndrome definitional criteria [33,34] and differences in genetic predisposition, various lifestyle patterns, nutritional behavior, as well as cultural differences [35,36,37,38].

It is also noteworthy that our study population was mainly women with a low socio-economic status, as more than one-third of them were petty traders, whose monthly incomes are less than $US 90. 

However, there was a high prevalence of risk factors for metabolic syndrome among the participants, similar to those obtained in Western countries. This may explain the comparable rate of metabolic syndrome observed in our study.

Our observed prevalence falls within the global prevalence of this phenomenon, which varies from 14.2% to 24.0% [37]. Reports from Africa have shown that metabolic syndrome is more common in females and increases with age and urban dwelling [32,37,39,40].

Our present study, which involved asymptomatic subjects, has demonstrated that, among the subjects with metabolic syndrome, 44 (95.7%) had 10-year cardiovascular risk <10% and only 2 (4.3%) had >10% risk. 

We observed that individuals with metabolic syndrome were significantly more likely to have increased FRS. None of the participants were in the high-risk category according to FRS. While many have reported FRS as a better predictor of coronary heart disease and stroke than metabolic syndrome [41], previous studies [41,42,43,44,45,46] have shown inconsistencies in the utility of metabolic syndrome and FRS for prediction of cardiovascular risk. It has yet to be determined whether one predicting tool is superior to the other in assessing cardiovascular risk among patients. However, both metabolic syndrome and FRS can be effectively used for predicting the long-term appearance of cardiovascular events, bearing in mind some of the potential limitations of each tool.

The findings from this study are consistent with other research, with respect to the prevalence of FRS. It is noteworthy that the majority of our study population was apparently normal individuals, with no history of smoking, although about 29% and 5.8% of them had family or personal history of hypertension and stroke, respectively.

Zarich *et al.* [47], in their assessment of the prevalence of metabolic syndrome and estimates of global risk using the Framingham Risk Score in young subjects with acute myocardial infarction, documented that two thirds of their subjects had metabolic syndrome. Their study also showed that 62% of the patients had the lowest Framingham risk category, and only 28% of subjects with MS had a Framingham Risk Score greater than 20%.

Yousefzadeh *et al.* [48] showed that 74.3% of their subjects with MetS were low-risk, 18.1% were intermediate-risk, and 7.6% were high risk for 10-year CVD. It was concluded in their report that there was a significant association between the presence of metabolic syndrome and a high risk for cardiovascular disease, based on FRS. In the same study, women with metabolic syndrome were found to have a higher risk of development of cardiovascular events compared to men. Similarly, a meta-analysis examining the association between the metabolic syndrome and risk of cardiovascular disease, conducted by Galassi *et al.* [49], showed that women were at higher risk of cardiovascular disease associated with the metabolic syndrome.

## 6. Conclusions

The prevalence of metabolic syndrome in these cohorts of semi-urban women was comparable to the global rate, despite their low economic status and lifestyles. Individuals with metabolic syndrome were significantly more likely to have cardiovascular disease than persons without metabolic syndrome, thus emphasizing the need for risk stratification and prompt management. As Nigeria is witnessing tremendous socio-economic changes and rural-urban migration, a call for urgent action to combat the growing epidemic of metabolic syndrome is advocated.

## 7. Limitations

The use of cross-sectional data could not help to examine the causal pathways and the generalization of the result obtained. Our data analysis was restricted to providing a snapshot of the prevalence of metabolic syndrome and evaluating cardiovascular risk among semi-urban women. The sample was not randomly selected, which could affect the actual prevalence of metabolic syndrome in the study population, albeit, the volunteers were asymptomatic. Selection bias might have accounted for the observed high prevalence of metabolic syndrome among these apparently healthy volunteers, as those who perceived they could be at risk were more likely to present themselves, having listened to the health-awareness talk earlier.

## Figures and Tables

**Table 1:** Socio-demography characteristic of the participants.

Characteristic	All Participants *N* (%)	Individuals with MetS *N* (%)	Individuals without MetS *N* (%)
**Occupation**			
House wife	6 (3.2)	-	6 (3.2)
Business	74 (39.2)	6 (3.2)	68 (36)
Civil servant	78 (41.3)	13 (6.9)	65 (34.4)
Farming	16 (8.5)	-	16 (8.5)
Clergy	15 (7.9)	-	15 (7.9)
**Average monthly income**			
<90 Dollars	114 (60.3)	8 (4.2)	106 (56.1)
90–250	36 (19.0)	3 (1.6)	33 (17.5)
251–500	35 (18.5)	8 (4.2)	27 (14.3)
>500	4 (2.1)	-	4 (2.1)
**Educational attainment**			
None	10 (5.3)	1 (0.5)	9 (4.8)
Primary	48 (25.4)	5 (2.6)	43 (22.8)
Secondary	61 (32.3)	6 (3.2)	55 (29.1)
Tertiary	59 (31.2)	5 (2.9)	54 (28.6)
Others	11 (5.8)	2 (1.1)	9 (4.8)

MetS = Metabolic syndrome.

**Table 2:** Clinical and laboratory parameters among the participants.

Characteristic	All Participants	Individuals with MetS	Individuals without MetS	*p* Value
Age	53.5	54.33 ± 10.55	53.24 ± 9.35	
Weight (kg)	70.12 ± 13.12	70.41 ± 12.47	70.02 ± 13.35	
BMI (kg/m^2^)	27.97 ± 4.79	26.98 ± 2.90	27.30 ± 6.83	
Waist hip ratio	0.87 ± 0.074	0.88 ± 0.059	0.86 ± 0.78	
Tchol (mmol/L)	5.43 ± 1.06	5.47 ± 0.78	5.41 ± 1.14	
TG (mmol/L)	1.14 ± 0.46	1.25 ± 0.43	1.11 ± 0.46	
HDLchol (mmol/L)	1.62 ± 0.47	1.50 ± 0.48	1.66 ± 0.47	
LDLchol (mmol/L)	3.28 ± 0.48	3.40 ± 0.70	3.23 ± 0.90	
**BP (mmHg)**				
Systolic	131.80 ± 30.17	152.39 ± 41.05	125.17 ± 22.13	
Diastolic	78.28 ± 13.58	85.54 ± 13.91	75.94 ± 12.66	
FBS (mmol)	5.40 ± 1.76	6.07 ± 2.82	5.18 ± 1.17	
**Categorical Data**				
FRS ≤ 10%	187 (98.9)	44 (23.3)	143 (75.7)	
FRS 10%–20%	2 (1.1)	2 (1.1)	-	0.012

MetS = metabolic syndrome; BMI = body mass index; Tchol = total cholesterol; HDLchol = high density cholesterol; LDLchol = low density cholesterol; FRS = Framingham risk score; FBS = fasting blood sugar.

## Abbreviations

The following abbreviations are used in this manuscript:

FRS	Framingham risk score
MetS	Metabolic syndrome
BMI	body mass index
Tchol	total cholesterol
HDLchol	high density cholesterol
LDLchol	low density cholesterol
CVD	cardiovascular disease
